# Immortalized porcine mesenchymal cells derived from nasal mucosa, lungs, lymph nodes, spleen and bone marrow retain their stemness properties and trigger the expression of siglec-1 in co-cultured blood monocytic cells

**DOI:** 10.1371/journal.pone.0186343

**Published:** 2017-10-16

**Authors:** Abubakar Garba, Lowiese M. B. Desmarets, Delphine D. Acar, Bert Devriendt, Hans J. Nauwynck

**Affiliations:** Department of Virology, Parasitology and Immunology, Faculty of Veterinary Medicine, Ghent University, Merelbeke, Belgium; Università degli Studi della Campania "Luigi Vanvitelli", ITALY

## Abstract

Mesenchymal stromal cells have been isolated from different sources. They are multipotent cells capable of differentiating into many different cell types, including osteocytes, chondrocytes and adipocytes. They possess a therapeutic potential in the management of immune disorders and the repair of damaged tissues. Previous work in our laboratory showed an increase of the percentages of CD172a^+^, CD14^+^, CD163^+^, Siglec-1^+^, CD4^+^ and CD8^+^ hematopoietic cells, when co-cultured with immortalized mesenchymal cells derived from bone marrow. The present work aimed to demonstrate the stemness properties of SV40-immortalized mesenchymal cells derived from nasal mucosa, lungs, spleen, lymph nodes and red bone marrow and their immunomodulatory effect on blood monocytes. Mesenchymal cells from nasal mucosa, lungs, spleen, lymph nodes and red bone marrow were isolated and successfully immortalized using simian virus 40 large T antigen (SV40LT) and later, co-cultured with blood monocytes, in order to examine their differentiation stage (expression of Siglec-1). Flow cytometric analysis revealed that the five mesenchymal cell lines were positive for mesenchymal cell markers CD105, CD44, CD90 and CD29, but lacked the expression of myeloid cell markers CD16 and CD11b. Growth analysis of the cells demonstrated that bone marrow derived-mesenchymal cells proliferated faster compared with those derived from the other tissues. All five mesenchymal cell lines co-cultured with blood monocytes for 1, 2 and 7 days triggered the expression of siglec-1 in the monocytes. In contrast, no siglec-1^+^ cells were observed in monocyte cultures without mesenchymal cell lines. Mesenchymal cells isolated from nasal mucosa, lungs, spleen, lymph nodes and bone marrow were successfully immortalized and these cell lines retained their stemness properties and displayed immunomodulatory effects on blood monocytes.

## Introduction

Mesenchymal stromal cells, also known as mesenchymal stem cells, are multipotent cells derived from the mesoderm during embryonic development [1, 2]. They have been demonstrated by many research groups to be a potential tool in treating cardio-vascular diseases, diabetes and autoimmune diseases, like rheumatoid arthritis as well as in regenerative medicine [3, 4, 5]. They have immunomodulatory properties, which they effect through many ways, one of which is the secretion of anti-inflammatory factors such as TGF-β [6]. They may inhibit the proliferation of lymphocytes and regulate the differentiation and function of dendritic cells [7]. Mesenchymal cell co-cultures with macrophages trigger an increase in the expression of IL-10 and decrease the expression of TNF-α and IL-12 [8]. *In vivo* experiments showed the accumulation of macrophages with a regulatory phenotype in inflamed areas upon local infusion of mesenchymal cells. The short life span of primary mesenchymal cells during *in vitro* cultivation prevents their use in long-term experiments [9, 10, 11]. Primary mesenchymal cells have a limited number of cellular divisions in cell culture after which they undergo senescence and finally die [12, 13]. Because of these limitations, there is an urgent need to establish continuous cell cultures of well-characterized mesenchymal cells for long-term studies. Presently, the most widely used method to immortalize primary cells is by introducing viral genes, such as the gene encoding simian virus 40 large T antigen [14, 15].

The ability to keep large quantities of mice for repetitive experiments makes it the most widely used animal for studying many human diseases and abnormalities. Many groups conducted research on the potential therapeutic application of mesenchymal stem cells in humans using mice models with successful outcome. However, its small size makes it impossible to collect large amounts of tissues for an experiment. Moreover, results obtained from experiments performed on mice may be difficult to successfully translate to human medicine [16].

Alternative large animal models may be developed with pigs, which are more closely related to humans than mice on an anatomical and physiological level [17]. Large amounts of tissues can be obtained from pigs to conduct several experiments. Siglec-1, a protein expressed only on macrophages, plays a crucial role in host-pathogen interactions and immune regulation. It mediates the receptor-dependent internalization of PRRSV [18]. Pathogens carrying sialic acids can be internalized by siglec-1^+^ macrophages [19]. In the present study, continuous cultures of mesenchymal cells from porcine nasal mucosa, lungs, spleen, lymph nodes and bone marrow were established and used to generate siglec-1^+^ macrophages.

## Materials and methods

### Cell isolation and cultures

Three pigs were euthanized by injecting sodium pentobarbital (20%, 1ml/1.5 kg; Kela Laboratories, Hoogstraten Belgium) into the jugular vein. The pigs were euthanized for the purpose of other experiments with the approval of Local Ethical and Animal Welfare Committee of the Faculty of Veterinary Medicine of Ghent University (Application EC2015∕04). Nasal mucosa, lungs, spleen and lymph nodes were removed in a sterile way and transferred immediately to a biosafety cabinet. Tissues from these organs were cut into small pieces, transferred into sterile 100 ml bottles containing Dulbecco’s Modified Eagle’s Medium (DMEM) and incubated at 37°C for 1 h in the presence of 0.5 mg/ml collagenase type IV (Gibco). Next, the cell suspension was filtered using a 70 μm cell strainer and washed two times with PBS. The cells were resuspended in DMEM supplemented with 10% fetal calf serum (FCS; Gibco), 1 mM sodium pyruvate, 1% non-essential amino acid, 0.1 mg/mL gentamicin (Invitrogen), 0.1 mg/mL streptomycin (Certa), and 100 U/mL penicillin (Continental Pharma). Cells were seeded in 24-well plates at a concentration of 1 x 10^6^ /ml. To deplete the cell cultures of mononuclear leukocytes, half of the medium was replaced every 48 hrs for 1 week. At 80% confluency, spindle and elongated-shaped cells were trypsinized in 0.125% trypsin solution (Sigma-Aldrich) and passaged. Isolation, cultivation, immortalization and characterization of red bone marrow mesenchymal cells were described before (Garba et al., 2017). To check the epithelial cell contamination in the immortalized mesenchymal cell cultures, at passage 11 the five immortalized mesenchymal cell types were seeded in 24-well plate and allowed to attach to a glass cover slide overnight, washed two times with PBS and fixed with 4% paraformaldehyde at room temperature for 10 minutes. The cells were washed with PBS and permeabilized with Triton-X 100 at room temperature for two minutes. Next, the cells were incubated with monoclonal mouse anti-human cytokeratin (AE1/AE3, 1:200, IgG1, Dako Denmark A∕S) for 1 h at 37°C in the presence of 10% normal goat serum. The cells were washed two times with PBS and later incubated with secondary antibodies goat anti-mouse IgG1-FITC for 1 h at 37°C. The cells were additionally washed two times with PBS and the cover slide was mounted with glycerin/PBS solution (0.9:0.1, v/v) and analyzed by confocal microscopy (Leica Microsystem DMRBE, Wetzlar, Germany). Also to detect the presence of mononuclear cells in the immortalized mesenchymal cell cultures. Cells were fixed with 4% paraformaldehyde for 10 min. at room temperature followed by permeabilization with Triton-X 100 for 2 min. at room temperature. The cells were washed two times with PBS and incubated with rabbit polyclonal antibodies to CD45 (ab10558, 1:100, IgG, abcam) for 1 h at 37°C in the presence of 10% normal goat serum. The cells were washed two times with PBS and further incubated with secondary antibodies goat anti-rabbit IgG-FITC for 1 h at 37°C. After two times washing with PBS, the cover slide was mounted with glycerin/PBS solution (0.9:0.1, v/v) and analyzed by confocal microscopy (Leica Microsystem DMRBE, Wetzlar, Germany). Mesenchymal cells derived from nasal mucosa, lungs, spleen and lymph nodes were monitored by light microscopy to examine their morphology.

### Immortalization of primary mesenchymal cells

To establish continuous cultures of mesenchymal cells for long-term studies, cells were immortalized using recombinant lentivirus containing the sequence encoding the simian virus 40 large T antigen (SV40LT) (Applied biological materials Inc., Richmond, BC, Canada). At 50% confluency, cells isolated from nasal mucosa, lungs, spleen and lymph nodes were incubated with an SV40LT gene carrying lentivirus suspension containing polybrene (8 μg/mL, applied biological material Inc., Richmond, BC, Canada). After 30 minutes, the virus was diluted with complete medium to prevent toxicity. Next, the cells were further incubated overnight. After 18 h incubation, the virus suspension was replaced with complete medium, after two times washing. The cells were allowed to proliferate for 2–3 days to 90–100% confluency. Three days post-transduction, cells were trypsinized with 0.125% trypsin (Sigma- Aldrich) and reseeded. The cells were examined every day for proliferation by light microscopy (Olympus). To confirm successful immortalization of mesenchymal cells, transduced cells were seeded in 24 well-plates on glass cover slips and incubated at 37°C, 5% CO_2_ for 24–48 h. Cells were washed two times with PBS and fixed with 4% paraformaldehyde for 10 min. at room temperature. Next, cells were additionally washed two times and permeabilized with Triton-X 100 at room temperature for two minutes. Subsequently, the cells were incubated with polyclonal rabbit antibodies against SV40LT (Applied Biological material Inc.) antigen for 1 h at 37°C in the presence of 10% normal goat serum. After two washes with PBS the cells were further incubated with goat anti-rabbit IgG-FITC for 1 h at 37°C. The cells were additionally washed two times with PBS and the cover slide was mounted with glycerin/PBS solution (0.9:0.1, v/v) with 2.5% 1,4-diazabicyclo [2.2.2] octane and analyzed by confocal microscopy (Leica Microsystem DMRBE, Wetzlar, Germany).

### Flow cytometry

Immortalized mesenchymal cells were analyzed for the expression of the mesenchymal cell markers CD44, CD29, CD105 and CD90. As a negative control, the expression of the myeloid cell markers CD16 and CD11b were also examined. Immortalized mesenchymal cells from nasal mucosa, lungs, spleen, lymph node and bone marrow cells were trypsinized with 0.125% trypsin (Sigma-Aldrich) and washed two times in PBS containing 1 mM EDTA-5% FCS. The cells were incubated with the following primary mouse monoclonal antibodies: anti-CD44 (F10-44-2, IgG2a, 1:200, abcam), anti-CD29 (7F10, IgG1, 1:200, THERMO Fischer Scientific Inc), anti-CD105 (MEM-229, IgG2a, 1:200, THERMO Fischer Scientific Inc.), anti-CD90 (3F9, IgG2a, 1:200, Norvus Biological), anti-CD16 (G-7, IgG1, I:100, Antigenix America Inc.) and anti-CD11b (ab8879, IgG1, 1:400, abcam). To demonstrate the specificity of the primary antibodies, 13D12 against gD of PrV (IgG1) and 1C11 against gB of PrV (IgG2a) were used as isotype controls (Nauwynck *et al*., 1995). The incubation was performed in the presence of normal goat serum for 30 minutes on ice. After two washes, the cells were incubated with appropriate secondary antibodies: Alexa Fluor 647-conjugated goat anti-mouse IgG2a and Alexa Fluor 488-conjugated goat anti-mouse IgG1 for 30 minutes on ice in the dark. Next, the cells were washed two times, resuspended in 100 μl PBS and transferred to 96 well-plates. The measurement of cells and analysis of the results were performed with a cytoFLEX flow cytometer and cytoFlex software respectively (Beckman Coulter, Inc.).

### Growth analysis of immortalized mesenchymal cells

To determine the proliferation rate of the immortalized mesenchymal cells, the cells were seeded in six-well plates at 2 x 10^5^/ml. Every 24 h, cells were trypisinized and counted during 5 days at 24, 48, 72, 96 and 120 h. The growth analyses were performed in triplicate at each time point.

### Cell cycle analysis of immortalized mesenchymal cells

Immortalized mesenchymal cells were trypsinized with 0.125% trypsin solution and washed two times in PBS. The cells were resuspended in ice-cold PBS and their concentrations adjusted to 10^6^ cells/ml. One ml of the cell suspension was gently vortexed and added dropwise to 9 ml of 70% ethanol in 15 ml centrifugation tubes. The cells were incubated at 4°C for 2 hours and then centrifuged at 450 x g at 4°C for 10 min. Subsequently, the cells were resuspended in 300 μl propidium iodide (1 mg/ml)/Triton X-100 (0.1%) solution containing 2 mg DNase-free RNase A (Sigma-Aldrich). Later, the cells were incubated at 37°C for 15 minutes in the dark. Next, the cells were transferred to 4°C until measurement. A CytoFLEX flow cytometer (Beckman Coulter, Inc.) was used to acquire and analyze the data.

### Osteogenic, chondrogenic and adipogenic differentiation of immortalized mesenchymal cells

Immortalized mesenchymal cells derived from nasal mucosa, lungs, spleen, lymph nodes and red bone marrow were resuspended in complete medium and seeded in 24-well plates at a concentration of 1 x 10^5^ cells/ml. The cells were incubated at 37°C in a humidified atmosphere of 5% CO_2_. At 60% confluency, medium was replaced with complete osteogenic differentiation medium (STEPRO^®^ osteogenesis Differentiation Kit; Gibco) and the cells were further incubated. The cell cultures were refed every 3 days. After 5 days of culturing, osteogenic differentiation medium was gently removed and mesenchymal cells were washed two times with PBS. The cells were fixed with 4% parafomaldehyde for 30 minutes at room temperature (RT). The cells were additionally washed two times with PBS and further incubated with 2% alizarin red solution for 3 minutes. After removal of the staining solution, cells were washed two times with PBS and analyzed by light microscopy. Adipogenic differentiation of the mesenchymal cells was performed by culturing mesenchymal cells in adipogenic differentiation medium (STEPRO® Adipogenesis Differentiation Kit; Gibco) for 5 days. To demonstrate the presence of lipid droplets in differentiated mesenchymal cells, adipogenic differentiation medium was removed. Next, the cells were washed two times with PBS and incubated with 4% paraformaldehyde for 30 minutes at room temperature. Later, 200 μl oil red O was added to the cells and incubated for 1 h at room temperature. The cells were washed two times with PBS and analyzed using light microscopy. For chondrogenesis, mesenchymal cells were cultured in chondrogenic differentiation medium (STEPRO^®^ Chondrogenesis Differentiation Kit; Gibco) for 7 days. The cell cultures were refed every 3 days. To confirm the presence of calcium, deposited on differentiated mesenchymal cells, cells were incubated with alcian blue staining solution for 30 minutes at RT. The cells were then washed two times with PBS and analyzed by light microscopy.

### Culturing immortalized mesenchymal cells in media supplemented with cytokine-free serum

After detachment, immortalized mesenchymal cells derived from nasal mucosa, lungs, spleen, lymph nodes and red bone marrow were suspended in DMEM supplemented with 10% cytokine-free serum, 0.1 mg/mL gentamicin (Invitrogen), 0.1 mg/mL streptomycin (Certa), and100 U/mL penicillin (Continental Pharma). The cells were seeded at 2 x 10^5^ cells/ml in 24-well plates and incubated in a humidified environment of 5% CO2 at 37°C. After 3 days of culture the cells were trypsinized using 0.125% trypsin and washed two times. Next, the cells were resuspended in 100 μl PBS in 96-well plates and incubated with 1 mg/ml propidium iodide (PI; Sigma-Aldrich) for 10 minutes on ice in the dark. Afterwards, the number of cells were measured using cytoFLEX.

### Co-culturing of immortalized mesenchymal cells and blood monocytes

#### Isolation of blood mononuclear cells

Blood was taken from three pigs, using 20 ml syringes with 18-gauge needles. The blood was transferred into a sterile 50 ml Falcon tube and diluted 1:1 with ice-cold PBS. Twenty milliliter of diluted blood was gently layered on top of 15 ml Ficoll-paque in a 50 ml tube and centrifuged at 450 x g for 45 min. at RT. The interphase band containing mononuclear cells was gently removed and transferred into a new Falcon tube. After centrifugation, the cells were suspended in lysis buffer and incubated on ice for ten minutes. The cells were washed two times with PBS and resuspended in RPMI/MEM (1:1) supplemented with 10% fetal calf serum (FCS), 1 mM sodium pyruvate, 1% non-essential amino acid, 0.1 mg/mL gentamicin (Invitrogen), 0.1 mg/1 mL streptomycin (Certa), and 100 U/mL penicillin (Continental Pharma). Next, the cells were seeded in 24-well plates on inserts at a concentration of 10^6^ cells/ml. The cells were incubated at 37°C, 5% CO_2_.

#### Co-culture of peripheral blood monocytes and immortalized mesenchymal cells

Eighteen hours post-seeding, non-adherent cells were gently removed by washing. Next, 3 x10^4^/ ml immortalized mesenchymal cells from nasal mucosa, lungs, spleen, lymph nodes and red bone marrow were added to the monocytic cell cultures in DMEM supplemented with 10% fetal calf serum (FCS), 1 mM sodium pyruvate, 1% non-essential amino acid, 0.1 mg/mLgentamicin (Invitrogen), 0.1 mg/mL streptomycin (Certa), and 100 U/mL penicillin (Continental Pharma). Wells with only monocytic cells were used as controls. The immortalized mesenchymal-monocytic cell co-cultures were incubated at 37°C, 5% CO2. The co-cultures were fixed with 4% paraformaldehyde for 10 minutes at RT at 24 h, 48 h and 1 week post-seeding. After permeabilization with 0.1% Triton-X100 at RT for 2 minutes, the cells were incubated with porcine anti-siglec-1 (41D3, IgG1, 1:5) or porcine anti-CD163 (2A10/11, IgG1, 1:200, AbD Serotec, Dusseldorf, Germany) primary antibodies containing normal goat serum for 1 h at 37°C. After two washes, the cells were incubated with goat anti-mouse IgG Alexa-Fluor 594 (A-21125, 1:400, invitrogen) secondary antibodies for 1 h at 37°C. Hoechst 33342 was used to stain the nuclei of the cells. Subsequently, the cells were washed two times with PBS. To demonstrate the specificity of the primary antibodies used, isotype-matched control monoclonal antibodies against gD of PRV (IgG1, 13D12) were used (Nauwynck *et al*., 1995). The cells were rinsed with PBS and mounted with glycerin/PBS solution (0.9:0.1, v/v) with 2.5% 1,4-diazabicyclo [2.2.2] octane. Next, the siglec-1 and CD163 positive cells were determined by counting a total of 500 cells per slide using fluorescence microscopy and photographed by confocal microscopy (Leica Microsystem DMRBE, Wetzlar, Germany). Subsequently, ImageJ software (U.S. National Institutes of Health, Bethesda, Maryland, USA) was used to calculate the fluorescence intensity of the siglec-1^+^ and CD163^+^ cells.

### Statistical analysis

Descriptive statistics was used to determine the difference in the percentage and expression of siglec-1 and CD163 by monocytic cells co-cultured with mesenchymal cells derived from nasal mucosa, lungs, spleen, lymph nodes and bone marrow.

## Results

### Immortalizing mesenchymal cells from different tissues does not affect their morphology

**Fig 1:**
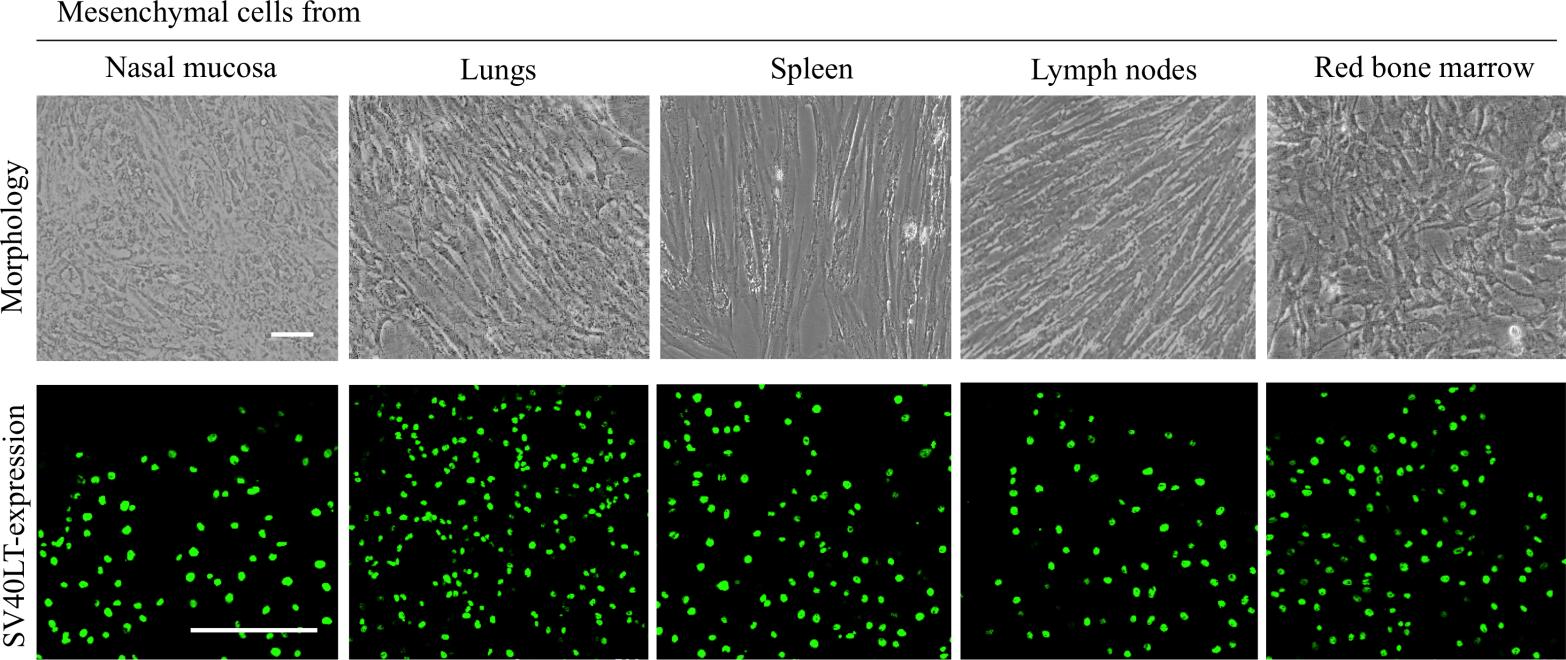
Morphological analysis of the immortalized mesenchymal cells. Top row: the morphology of the immortalized mesenchymal cells. Scale bar ꞊꞊ 10 μm. Bottom row: mesenchymal cells expressed nuclear localized SV40TL antigen. Scale bar ꞊꞊ 50 μm.

Seventy-two hours after seeding, a variety of cells attached to the culture plates. The cells included mononuclear cells, epithelial-like cells and elongated fibroblast-like cells. Passaging the cells depleted the epithelial-like cells and mononuclear cells, leaving fibroblast-like cells in culture which continued to proliferate. This was confirmed by staining the five immortalized mesenchymal cell types with antibodies against the epithelial cell marker cytokeratin and leukocyte marker CD45. No cytokeratin positive cells were found and no CD45 positive cells were observed in all the five immortalized mesenchymal cell cultures. The mesenchymal cells from nasal mucosa, lungs, spleen and lymph nodes were confirmed to be successfully immortalized by the expression of SV40LT antigens (Fig 1). The morphology of the immortalized cells was similar to that of the primary cells. All five mesenchymal cell lines showed a spindle-shape morphology (Fig 1).

The mesenchymal cells from nasal mucosa, lungs, spleen, lymph node and red bone marrow have been passaged 32 times without any problem regarding their morphology and growth characteristics.

### The mesenchymal cell specific marker profile is not affected by immortalization

**Fig 2:**
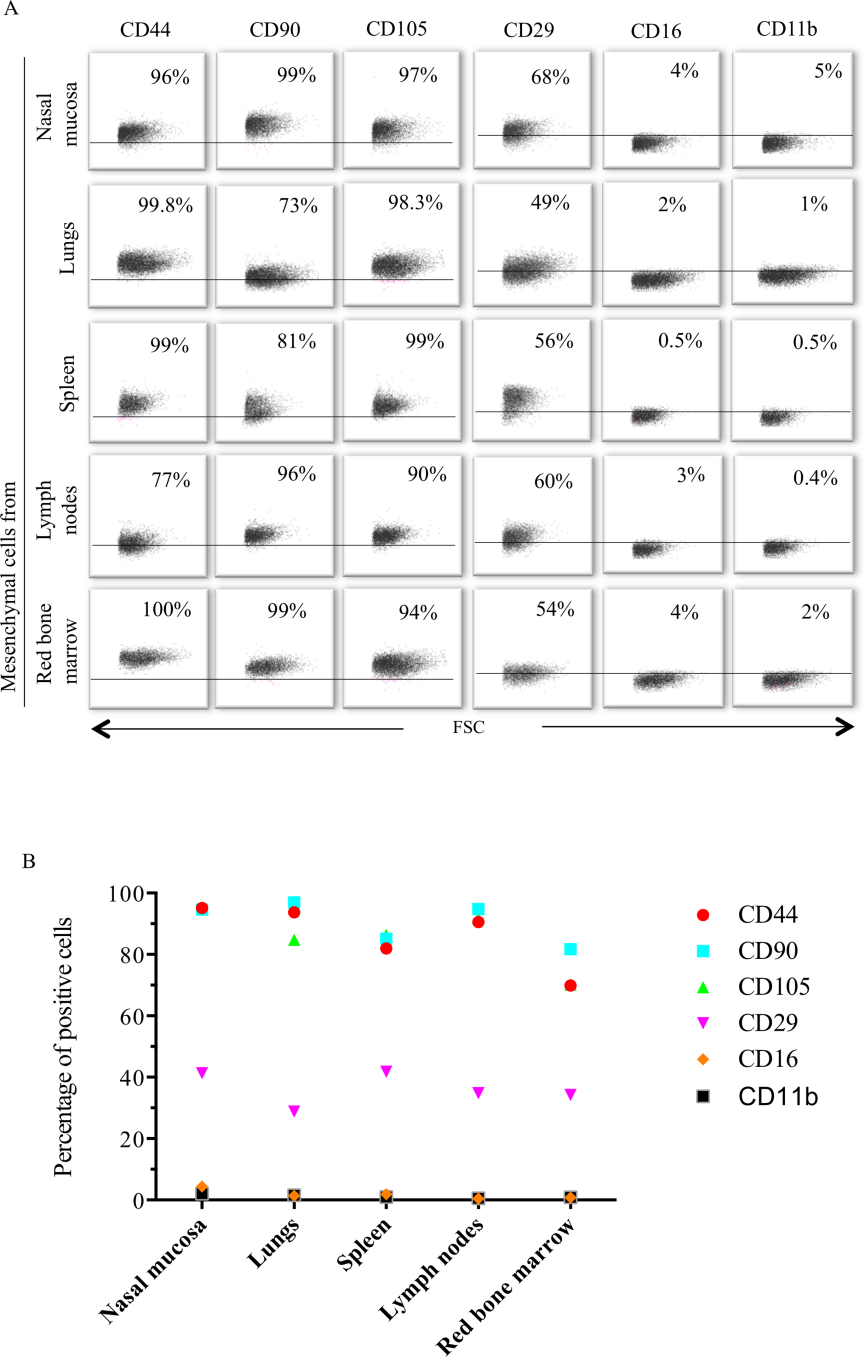
Characterization of immortalized mesenchymal cells by flow cytometry. Immunophenotyping was performed for detection of specific cell markers known to be expressed by mesenchymal cells (CD44, CD105, CD90 and CD29) and non-mesenchymal cell markers (CD16 and CD11b) (A). Three independent experiment (B). The percentages of CD105^+^, CD90^+^, CD44^+^ and CD29^+^ immortalized mesenchymal cells were 97±3%, 94±4%, 96±3% and 42±9%, respectively, in cells from nasal mucosa; 84±11%, 96±3%, 260 93.8±% and 29±3%, respectively, in cells from lungs; 86±6%, 85±3%, 81±1% and 41±2%, respectively, in cells from spleen; 92±0.5%, 95±0.6%, 91±2% and 34±2%, respectively, in cells from lymph nodes; 70±11%, 82±5%, 70±4% and 34±2%, respectively, in cells from red bone marrow.

Immortalized mesenchymal cells derived from nasal mucosa, lungs, lymph nodes, spleen and bone marrow were analyzed for the expression of mesenchymal cell specific markers by flow cytometry. The five mesenchymal cell lines expressed CD105, CD90, CD44 and CD29, but were negative for CD16 and CD11b (Fig 2A and 2B).

### Immortalized mesenchymal cells from different tissues display equal growth curves

**Fig 3:**
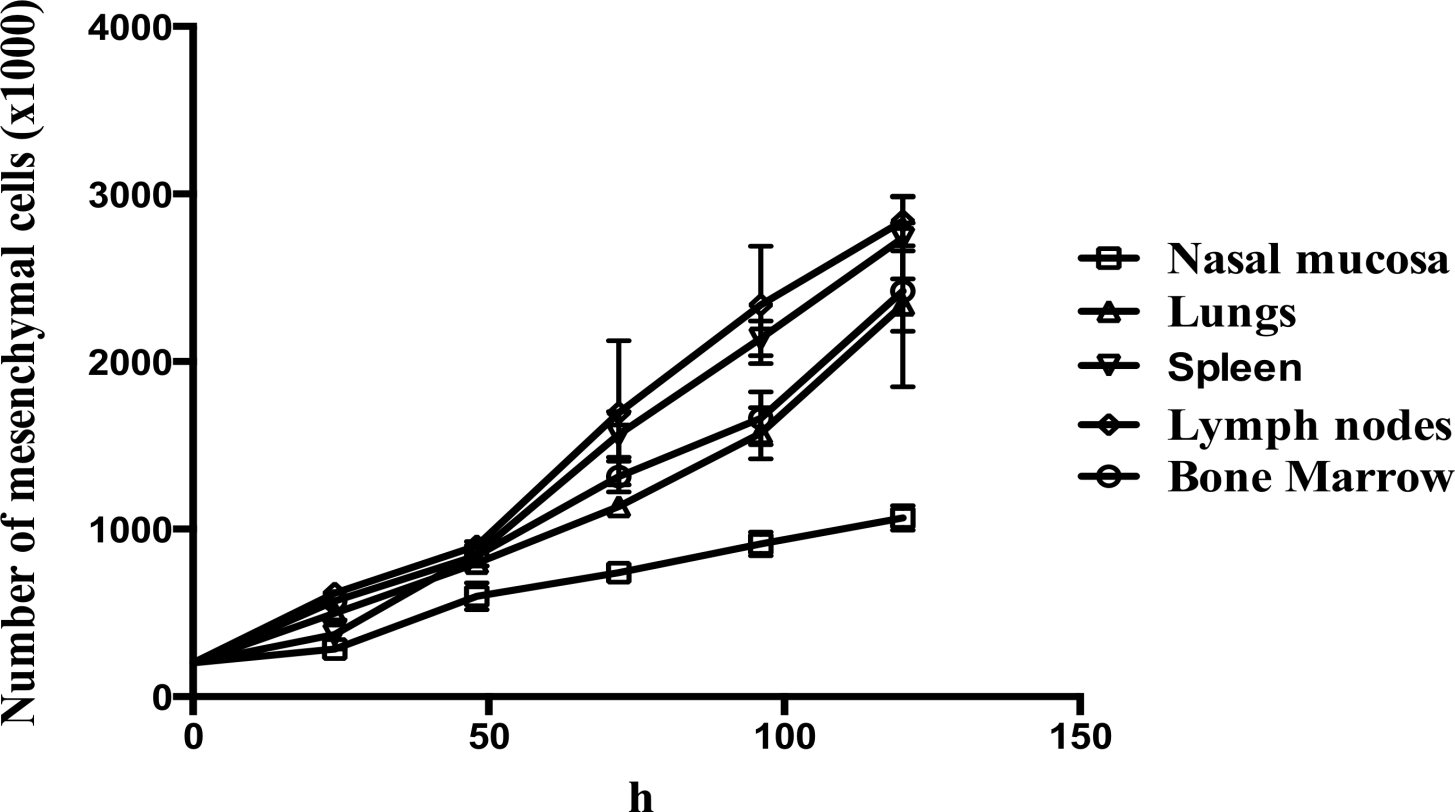
Proliferation analysis of the immortalized mesenchymal cells. Growth pattern of the five immortalized mesenchymal cells. The data are represented as mean +/- SD of three independent experiments.

The five immortalized mesenchymal cell types showed a similar growth pattern especially at the lag phase of the growth curve (Fig 3).

Five hours after seeding, 90% of the immortalized mesenchymal cells derived from lungs, spleen and lymph nodes attached to the bottom of the culture plates and started to spread. Only 50% of the nasal mucosa mesenchymal cells attached to the plate 5 hours post-seeding. In addition, the mesenchymal cells from the nasal mucosa not only attached slower to the bottom of the culture plate, they also proliferated slower when compared to the other cells.

### Replication capabilities of the immortalized mesenchymal cells

**Fig 4:**
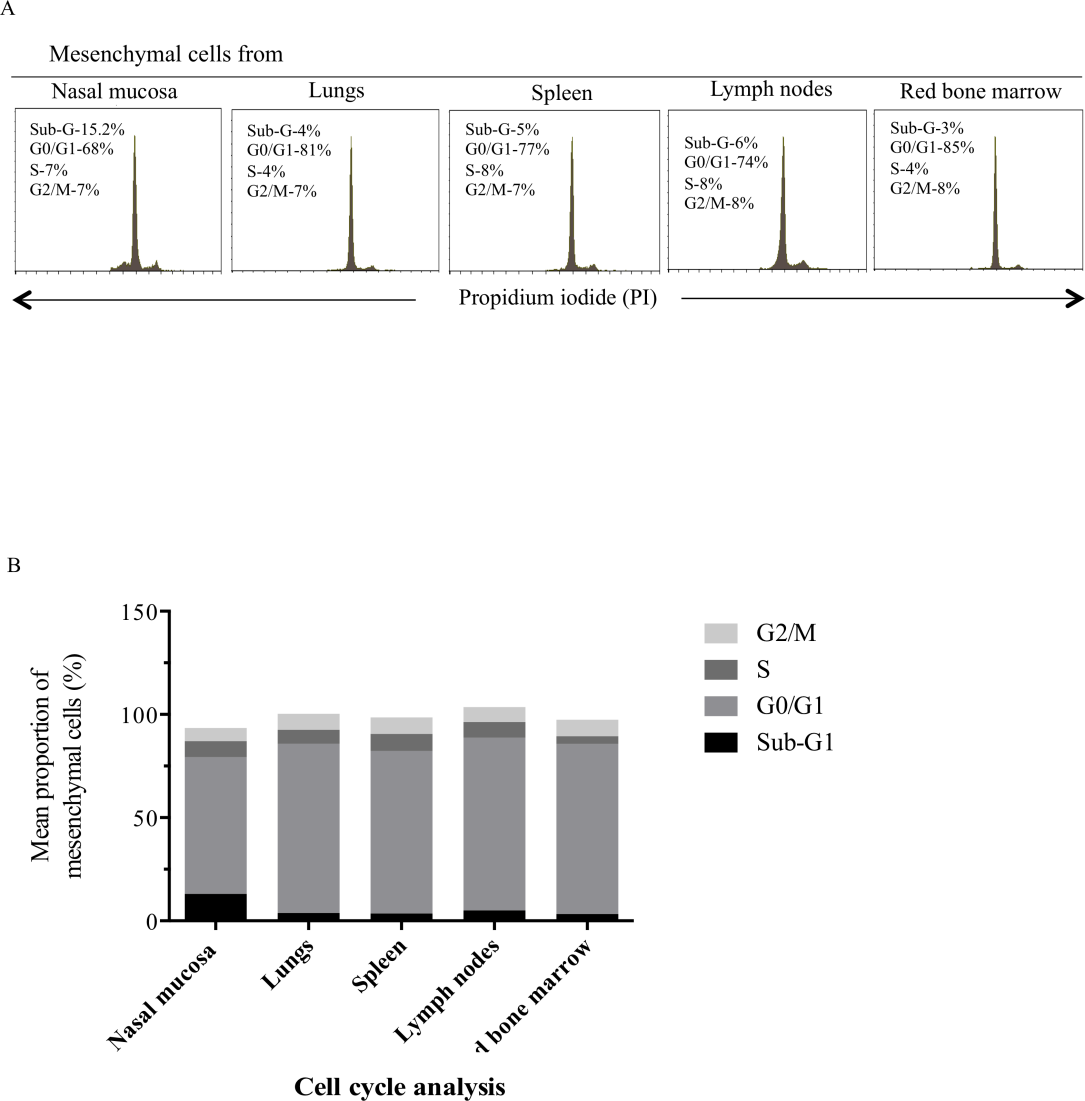
Cell cycle analysis of immortalized mesenchymal cells. Most cells of the five mesenchymal cells were at the G0/G1 phase of cell cycle (A). The data are represented as mean of three independent experiments (B).

Cell cycle analysis was performed to distinguish immortalized mesenchymal cells in different phases of the cell cycle for determining their replicative capabilities. It was observed that the majority of the cells were at the G0/G1 phase of the cell cycle. More mesenchymal cells were at sub-G1 phase of the cell cycle when originated from nasal mucosa than from the other tissues. The percentages of nasal mucosa, lung, spleen, lymph node and red bone marrow mesenchymal cells that were at the S phase of the cell cycle were 8±0.5%, 7±0.4%, 8±0.6%, 8±0.6%, 4±0.3%, respectively; 6±1%, 8±0.5%, 8±0.1%, 7±0.6%, 8±0.06% were at the G2/M phase, respectively (Fig 4A and 4B).

### Immortalized mesenchymal cells retain the ability to differentiate to osteo-, chondro- and adipocytes

**Fig 5:**
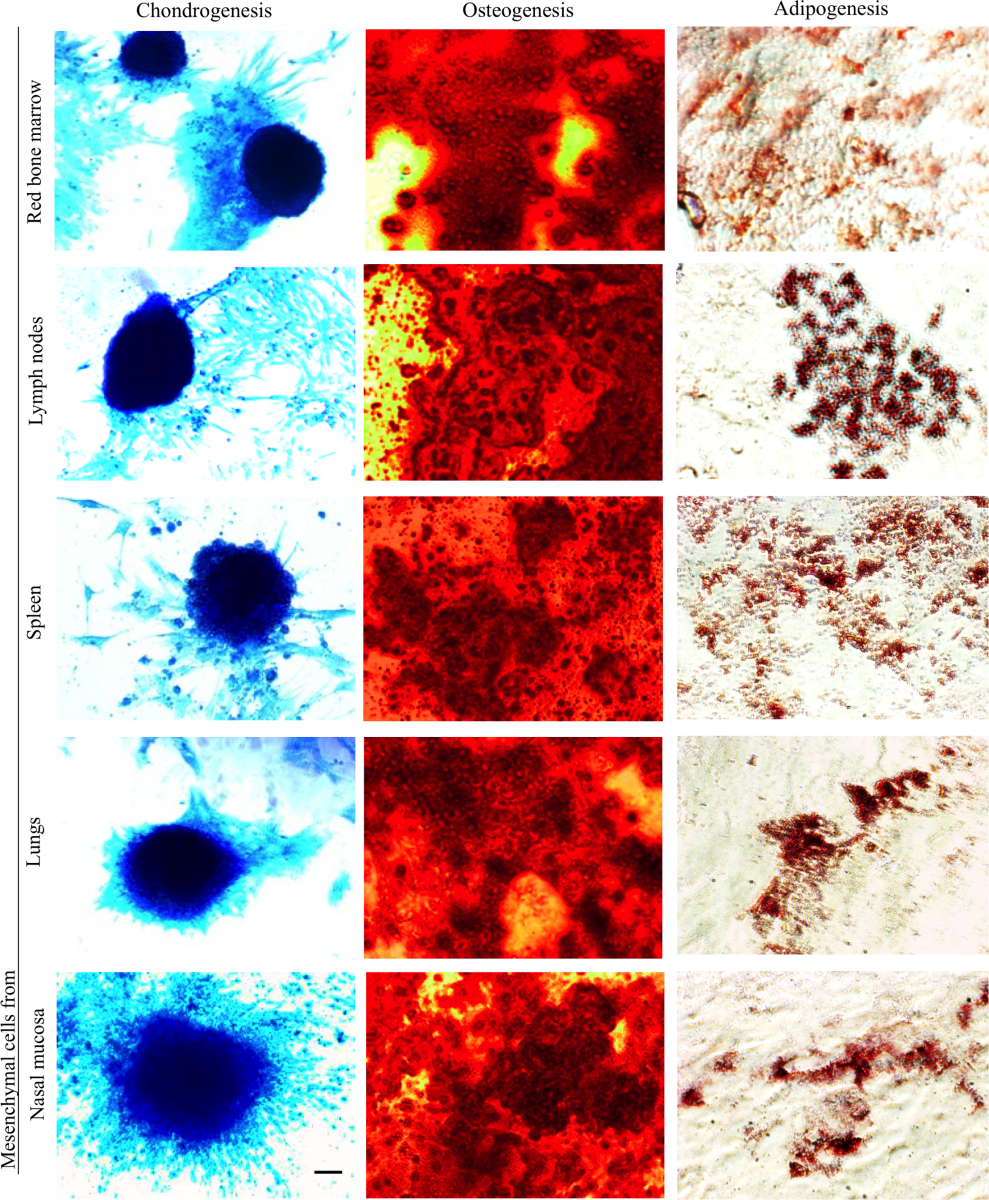
*In vitro* differentiation of immortalized mesenchymal cells. Immortalized mesenchymal cells differentiation into chondrocytes (see dark-blue color-chondrocytes), osteoclast (see red color-calcium deposit), and adipocytes (see lipid droplets in the cells) confirmed by staining with alcian blue, alizarin red and oil red, respectively. Scale bar ꞊꞊ 10 μm.

Five days after incubation with osteogenic differentiation medium, the immortalized mesenchymal cells were differentiated into osteoclasts as demonstrated by staining with alizarin red (an organic compound which binds specifically to calcium ions). Deposits of calcium (red) were observed with the five different mesenchymal cell lines (Fig 5).

100% of the immortalized mesenchymal cells from nasal mucosa, spleen, lymph nodes and red bone marrow and 70–80% of the lung-derived immortalized mesenchymal cells differentiated into osteocytes. After culturing the immortalized mesenchymal cells in adipogenic differentiation medium for 5 days, adipogenesis was demonstrated by staining with oil red O solution. Lipid droplets were observed on all differentiated mesenchymal cells (Fig 5). To confirm the differentiation of mesenchymal cells derived from nasal mucosa, lungs, lymph nodes, spleen and red bone marrow into chondrocytes, immortalized mesenchymal cells were incubated in chondrocyte differentiation medium. After 7 days, the mesenchymal cells showed a dark-blue coloration upon alcian blue staining, indicating that the mesenchymal cells were differentiated into chondrocytes (Fig 5). 50–60% of the red bone marrow-derived immortalized mesenchymal cells, 70–80% of the nasal mucosa and lymph node-derived immortalized mesenchymal cells and 100% of lung and spleen-derived mesenchymal cells differentiated into chondrocytes after 1 week of culture in chondrogenic differentiation medium.

### Viability of immortalized mesenchymal cells cultured in cytokine-free serum

**Fig 6:**
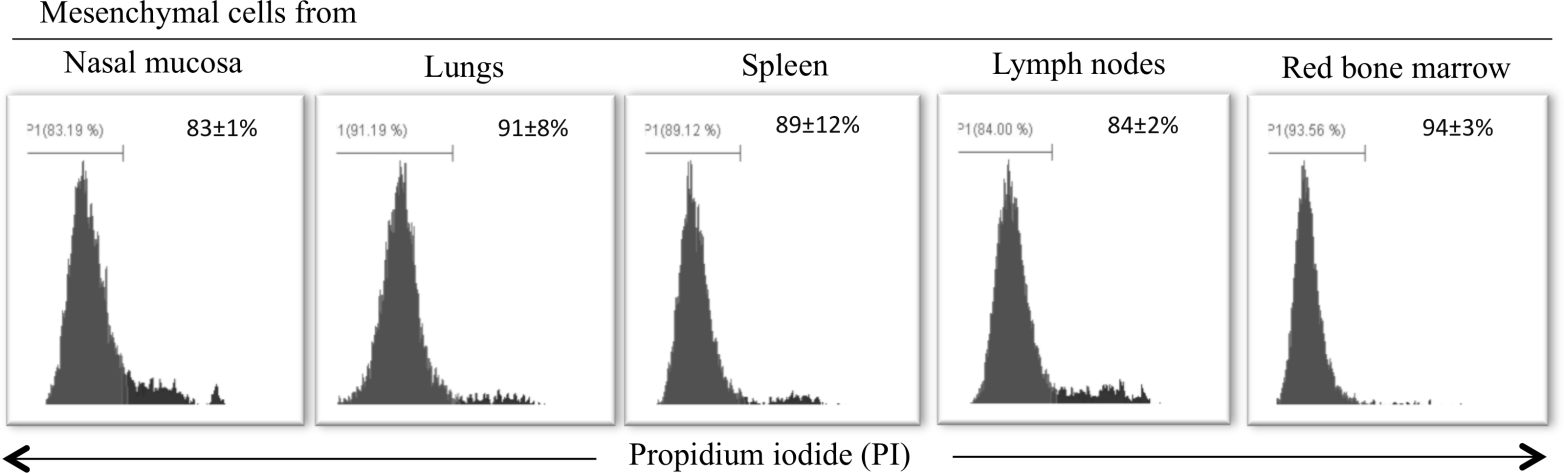
Expansion of immortalized mesenchymal cells in medium supplemented with cytokine-free serum. Viability of immortalized mesenchymal cells derived from nasal mucosa, lungs, spleen, lymph nodes and bone marrow cultured in DMEM supplemented with cytokine-free serum. The data are represent as mean +/- SD of three independent experiments.

To develop a culture system for accessing the immunomodulatory effect of the mesenchymal cells without interference of cytokines present in the fetal calf serum, immortalized mesenchymal cells were cultured in DMEM supplemented with cytokine-free serum for one week and their viability was determined afterwards. Flow cytometry data analysis indicated 83±1%, 91±8%, 89±12%, 84±2% and 94±3% viable cells in cultures of mesenchymal cells from nasal mucosa, lungs, spleen, lymph nodes and red bone marrow, respectively, after 7 days of culture (Fig 6).

### Immortalized mesenchymal cells induced the expression of siglec-1 in co-cultured blood monocytes

**Fig 7:**
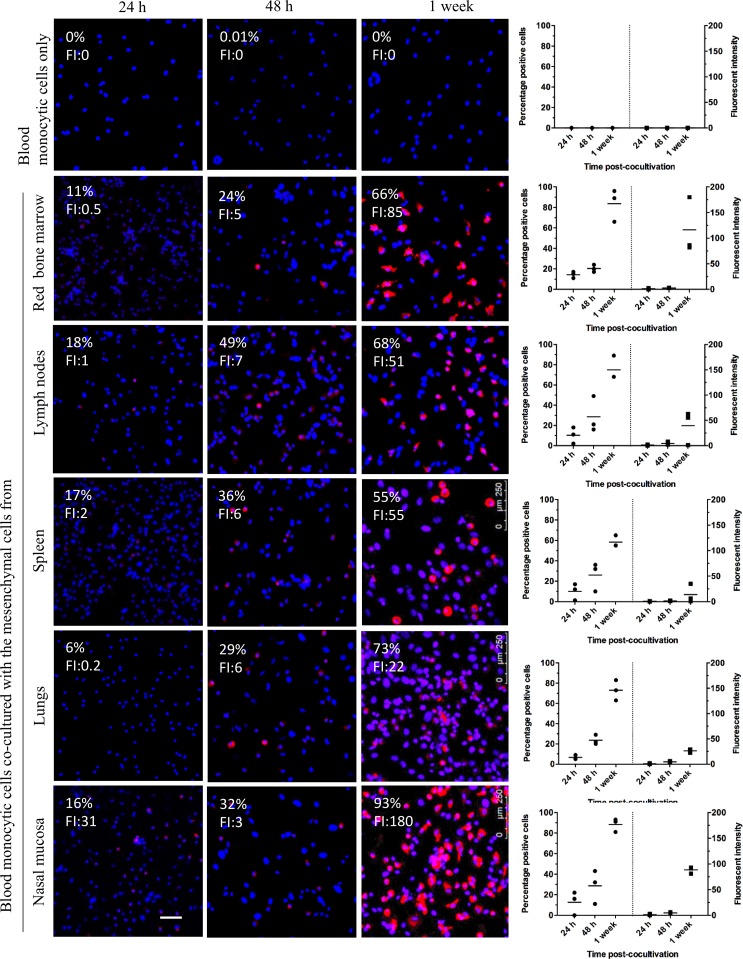
Co-culturing blood monocytic cells with immortalized mesenchymal cells triggers siglec-1 expression. Each point represents an individual pig (three independent experiments). FI: fluorescent intensity.

Nasal mucosa, lung, spleen, lymph node and bone marrow derived immortalized mesenchymal cells were co-cultured with blood monocytes in DMEM supplemented with 10% FCS. An increase in the percentages of siglec-1 positive monocytes and its expression from 24 hours to one week after the start of co-culture was observed: from 16% to 93% (nasal mucosa-derived mesenchymal cells), from 6% to 73% (lung-derived mesenchymal cells), from 17% to 55% (spleen-derived mesenchymal cells), from 18% to 68% (lymph node-derived mesenchymal cells) and from 11% to 66% (red bone marrow derived-mesenchymal cells). Siglec-1 was not expressed in monocytic cells cultured without mesenchymal cells (Fig 7).

**Fig 8:**
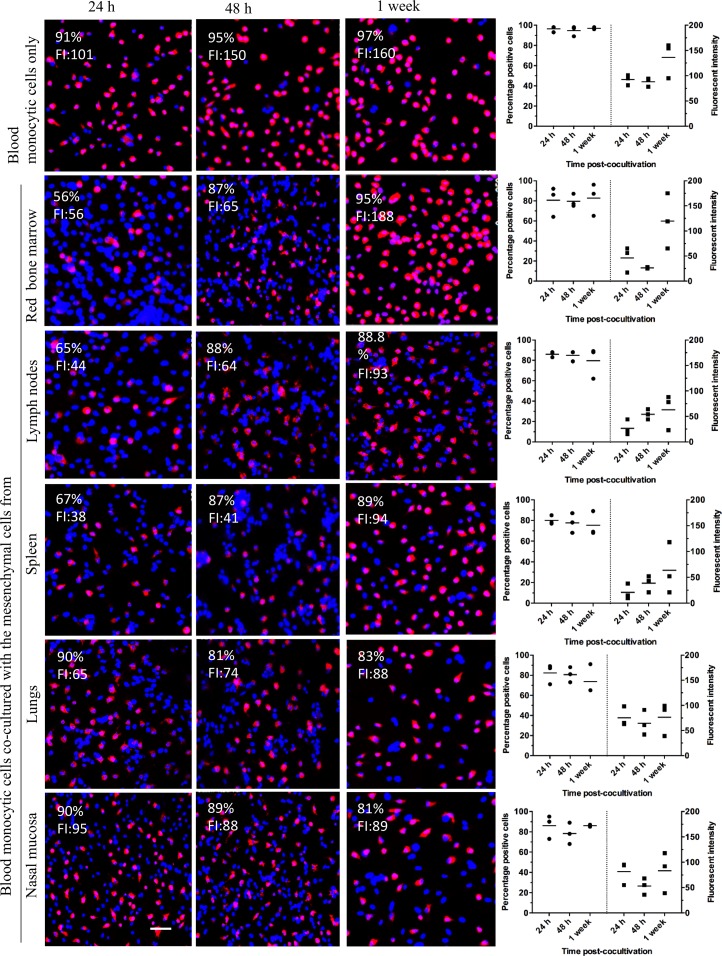
Co-culturing blood monocytic cells with immortalized mesenchymal cells. CD163 expression. Each point represents an individual pig (three independent experiments). FI: fluorescent intensity.

In contrast to siglec-1, the results indicated some variations in the expression of CD163 especially at 24 h after the start of the co-culture. There was a decrease in the percentages of CD163^+^ blood monocytic cells co-cultured with immortalized spleen, lymph nodes and bone marrow derived-mesenchymal cells when compared with pure blood monocytes (in the absence of mesenchymal cells). Also at 48 h and one week after the start of co-culture a slight decrease in the percentages of CD163^+^ cells was observed when compared with monocytes cultured without mesenchymal cells. Similarly, the fluorescence intensity of CD163^+^ in blood monocytic cells co-cultured with mesenchymal cells decreased when compared with monocytes cultured without mesenchymal cells. Variation among the three individual pigs used in this study was observed (Fig 8).

## Discussion

Mesenchymal cells have already been isolated and characterized from different tissues and organs of humans, mice and other animals [20, 21, 22, 23]. Isolated mesenchymal cells undergo senescence or apoptosis by passaging in *in vitro* cultures. To be able to expand the cells continuously and generate large amounts of mesenchymal cells for long-term research, such as tissue engineering, mesenchymal cells were immortalized using a wide range of methods [24, 25]. In this study, mesenchymal cells derived from nasal mucosa, lungs, spleen, lymph nodes and red bone marrow were successfully isolated, immortalized and characterized. We demonstrated the ability of these cells to trigger the expression of siglec-1 by blood monocytic cells during their co-culture. Results from this work indicated that mesenchymal cells derived from nasal mucosa, lungs, spleen, lymph nodes and red bone marrow expressed the mesenchymal cell specific markers CD105, CD90 and CD44, but were negative for CD16 and CD11b. Similar results were reported by many research groups for human, mice and porcine mesenchymal stem cells [26, 27, 28]. The five immortalized mesenchymal cell lines continued to grow without undergoing senescence after several passages, while the primary mesenchymal cells died after some passages. It was reported that human and guinea pig primary mesenchymal cell growth and proliferation rates decreased gradually after a few passages [29]. A growth curve analysis demonstrated that the five immortalized mesenchymal cell lines had a similar growth pattern, with red bone marrow-derived mesenchymal being the strongest grower and nasal mucosa-derived mesenchymal cells being the slowest grower. This was confirmed by cell cycle analysis which showed that a substantial percentage of immortalized nasal mucosa derived mesenchymal cells were in the sub-G phase, which are considered to be dead cells. In this study, the immortalized mesenchymal cells could be differentiated into osteocytes, chondrocytes and adipocytes [30, 31, 32]. Interestingly, all five mesenchymal cell types required only 4 days to differentiate into osteocytes and adipocytes and 7 days to differentiate into chondrocytes after initial culture in osteogenic, adipogenic and chondrogenic differentiation medium, respectively. This indicates that mesenchymal cell cultures established in this work need only a short time to differentiate into osteoclasts, adipocytes and chondrocytes. Indeed, many reports mentioned that mesenchymal cells of humans and mice origin need two to three weeks to differentiate into osteocytes, adipogenic and chondrocytes after initial exposure to specific differentiation medium [33].

Siglec-1, also called sialoadhesin or CD169, is a member of the sialic acid binding immunoglobulin-like lectin family found to be expressed on differentiated tissue macrophages in lungs, liver, colon, spleen, lymph nodes and bone marrow [34]. Siglec-1 has been reported to function in cell-cell interactions and in receptor-dependent internalization processes such as the internalization of porcine reproductive and respiratory syndrome virus (PRRSV) [35]. It is not expressed on porcine monocytes.

The majority of research groups that work with human, mice and other animal model mesenchymal stem cells use T-lymphocytes, B-lymphocytes, NK cells and dendritic cells to assess the immunomodulatory effect of mesenchymal cells with little emphasis given to cells of the monocyte/macrophage lineage [36, 37, 38]. Previous findings indicated functional interactions between macrophages and mesenchymal cells leading to phenotypic changes in the macrophages [39]. In the present study, the effect of mesenchymal cells on the differentiation of blood monocytes was examined. SV40LT immortalized mesenchymal cells from nasal mucosa, lungs, spleen, lymph nodes and red bone marrow triggered the expression of the macrophage differentiation marker siglec-1 in co-cultured blood monocytic cells.

A higher percentage of siglec-1^+^ monocytic cells was observed when co-cultured with immortalized mesenchymal cells derived from the nasal mucosa.

## Conclusions

In conclusion, SV40LT immortalized mesenchymal cells derived from nasal mucosa, lungs, spleen, lymph nodes and red bone marrow were successfully established and differentiated into osteocytes, chondrocytes and adipocytes. Moreover, they were able to activate the expression of siglec-1 on blood monocytes. This method can now be used to establish continuous mesenchymal stem cell lines from different tissues for long-term research such as tissue engineering. It can also be used to generate large amounts of siglec-1^+^ macrophages in a few days in a simple and affordable culture system for use in laboratory and clinical settings.
